# Clinical characteristics, lung function and airway inflammatory patterns of Brazilian children with severe therapy-resistant asthma

**DOI:** 10.1186/2045-7022-5-S2-P5

**Published:** 2015-03-23

**Authors:** Rodrigues Andrea, Giovana Santos, Rodrigo Souza, Mauro Vargas, Cristian Roncada, Leonardo Pinto, Marcus Jones, Renato Stein, Paulo Pitrez

**Affiliations:** 1 PUCRS, Pediatrics, Porto Alegre, Brazil

## Background

Severe therapy-resistant asthma (STRA) in children has been poorly studied, with mechanisms of disease remaining unclear. The aim of the study is to describe the clinical characteristics, lung function and lower airways inflammation in Brazilian children with STRA, comparing with mild asthmatics and healthy controls.

## Method

Children with STRA from a follow-up reference centre, and mild asthmatics and healthy controls from a cross-sectional study from public schools, were selected. Clinical characteristics, spirometry reports and induced sputum results were collected and compared from study databases.

## Results

20 children with STRA (mean age: 11.3±2.9 years; 62% males) were included, and paired with 70 children with mild asthma and 27 healthy controls. 18/20 (90%) STRA children were atopic, and only 2/20 (10%) were sensitized to pets. Lung function from children with STRA was not different from the other groups studied. From 13 STRA children with induced sputum obtained, we have found seven, four and two neutrophilic, eosinophilic and paucigranulocytic patterns, respectively. The number/percentage of inflammatory cells and pattern of sputum inflammation were not different between children with STRA (n=13) and milder asthma (n=70). From 5 sputums repeated in STRA children, 4 (80%) had the inflammatory pattern changed. Six STRA patients are under omalizumab treatment.

## Conclusion

Children with STRA have nearly normal lung function and their airway inflammatory pattern seems not to be different from children with milder asthma. The mechanisms involved in the uncontrolled disease of children with STRA are not clear and should be better addressed in future studies.

